# “Who can I ring? Where can I go?” Living with advanced cancer whilst navigating the health system: a qualitative study

**DOI:** 10.1007/s00520-022-07107-1

**Published:** 2022-05-10

**Authors:** Jade C. Newton, Moira O’Connor, Christobel Saunders, Sayed Ali, Anna K. Nowak, Georgia K. B. Halkett

**Affiliations:** 1 Faculty of Health Sciences, Curtin School of Nursing, Curtin University, GPO Box U1987, Perth, WA 6845 Australia; 2 WA Cancer Prevention Research Unit (WACPRU), School of Population Health, Faculty of Health Sciences, Curtin University, GPO Box U1987, Perth, WA 6845 Australia; 3 Medical School, The University of Western Australia, 35 Stirling Highway, Perth, WA 6009 Australia; 4 Medical Oncology Department, St John of God Midland Public Hospital, 1 Clayton Street, Midland, WA 6056 Australia; 5 Department of Medical Oncology, Sir Charles Gairdner Hospital, Hospital Avenue, Nedlands, WA 6009 Australia

**Keywords:** Neoplasms, Patient-centred care, Qualitative research, Continuity of patient care, Multidisciplinary communication

## Abstract

**Background:**

People with advanced cancer often experience greater physical and psychosocial morbidity compared to those with early disease. Limited research has focused on their experiences within the Australian health system. The aim of this study was to explore the lived experiences of adults receiving care for advanced cancer.

**Methods:**

A qualitative design with a descriptive phenomenological approach was used to explore the lived experiences of people with advanced cancer following their diagnosis. Twenty-three people living with an advanced solid malignancy receiving care were referred by their oncologists to take part in an interview conducted at their home, the hospital, or over the phone.

**Results:**

Three key themes emerged relating to participants’ experiences of living with advanced cancer: (1) living with a life-limiting diagnosis and uncertainty, (2) living with symptom burden and side effects, and (3) living within the health system, with two subthemes, the patient-clinician relationship, and care coordination. Participant relationships with their health professionals were particularly important and had a defining impact on whether patient experiences living with cancer were positive or negative.

**Conclusion:**

People with advanced cancer experienced broad variation in their experiences navigating the health system, and their relationships with clinicians and other health professionals were important factors affecting their perceptions of their experiences. Attention to the coordination of care for people with advanced cancer is necessary to improve their experiences and improve symptom control and the management of their psychosocial burden.

**Supplementary Information:**

The online version contains supplementary material available at 10.1007/s00520-022-07107-1.

## Introduction

Whilst clinical advances allow more people to survive a cancer diagnosis, [1, 2] many cancers are detected at a later stage of the disease where treatment goals are palliative as the diagnosis is incurable. An advanced cancer diagnosis has been recognised as a risk factor for anxiety and depression [3–5], which is concerning as psychosocial morbidities may result in treatment non-adherence, poorer quality of life, longer inpatient episodes, and negative impacts on family [6]. Providing optimal care is essential to ensure patients with life-limiting diagnoses are able to experience optimal quality of life.

### Living with advanced cancer

Systematic reviews in this area have identified that living with advanced cancer is a complex process, affected by an individual’s support network, personal coping strategies, and attitudes towards their diagnosis [7, 8]. Garcia-Rueda et al. [7] found that ‘health context’ was another prominent theme, which encompasses the ‘hospitalisation experience’ and their ‘experience of healthcare access’.

### The health context

Garcia-Rueda et al. [7] found that patient-clinician relationships are important elements of patient experiences within the health system. These could be targeted to improve patient quality of life by providing personalised care that acknowledges the unique challenges faced by people with life-limiting illnesses [9–14]. These findings have subsequently been built upon by an increase in qualitative research that explores patient perceptions of early palliative care interventions, seeking to address psychosocial distress people with advanced cancer may experience [15–21]. These studies have been undertaken predominantly in the USA and in European settings, and consistently identify coordination of care to be an issue in health systems where patient care involves multiple services.

Few qualitative studies have explored the lived experiences of Australians with advanced cancer. In 2018, Chambers et al. [22] found that the experiences of men living with advanced prostate cancer were mediated by their life course and social and health context, and identified a need for holistic approaches to care to address issues related to patient-clinician communication and care coordination. An understanding of patient experiences navigating the health system provides a foundation for understanding how existing resources are meeting patient needs, and identifying areas for improvement.

To our knowledge, there have been no studies examining the experiences of people living with advanced cancer in Western Australia. The health context for cancer care in Western Australia is presented in Table 1. Understanding the role the health system plays in the experiences of people with advanced cancer is essential to identify and address priority issues. This study aimed to explore the lived experiences of people with advanced cancer, focussing on their interactions with the health system and health professionals.

**Table 1:** The health context in Western Australia: coordination of cancer care

Component of cancer care	Description
Cancer treatment	Provided at tertiary and secondary hospitals, located in the state and territory capital cities Some densely populated outer-metropolitan suburbs and large rural towns are also equipped with specialised cancer units [41, 42]. Whilst cancer treatment is provided at all major hospitals, other illnesses have specialists located only at certain hospitals, so cancer patients with more complex comorbidities may need to attend multiple hospitals for specialised care
Cancer care coordination	The organisation of care coordination differs between states and services [43]. In order to help coordinate care in Western Australia, the state government manages a Cancer Nurse Coordination service to assist Western Australians with navigating the health system [44]

## Materials and methods

### Design

A descriptive phenomenological approach was used to explore the lived experiences of people with advanced cancer [23]. Van Manen [24] describes phenomenology as a means to interpret, understand, and reflect on participants’ lived experiences that facilitates a more complete understanding of what patients go through. Methods for recruitment and data collection are described elsewhere [25].

### Recruitment

Adults attending a medical oncology appointment relating to an advanced solid tumour diagnosis (advanced cancer defined as stage IV, metastatic, or recurrent, or receiving palliative care) were invited by their oncologist to take part in the study. Participants who gave verbal consent to be contacted were followed up by a researcher, who obtained written informed consent and arranged an audio-recorded interview. People receiving care for a haematological malignancy or those with cognitive impairment, defined as a score less than 7 on the Abbreviated Mental Test, were ineligible. The researcher administered the Abbreviated Mental Test before the interview. Haematological malignancies were excluded because treatment and disease progression are different from solid tumour malignancies [26]. A purposive sampling strategy was used (Table 2), and recruitment occurred until inductive thematic saturation was reached across the data.[27], defined as when no new themes arose within the interviews. Analysis occurred concurrently to the interviews to identify when no new themes arose from the interviews.

**Table 2:** Steps taken to establish rigour in qualitative research [29]

Bradshaw et al.’s criteria of rigour	Activities undertaken
Credibility	**Establish rapport** The interviewer initially contacted participants via phone or email to arrange interviews, providing an opportunity to develop rapport. Additionally, during the interviews, the interviewer favoured developing rapport and maintaining a conversational interview rather than sticking strictly to the interview guide. The interviewer used interview prompts and showed empathy which assisted participants to feel comfortable and freely discuss their experiences **Member checking** As participants were receiving care for an advanced diagnosis, we did not invite them to verify their manuscript transcriptions. However, at the end of each interview, the interviewer summarised what had been discussed and was given the opportunity to clarify any discrepancies or expand on any aspects of their interview
Confirmability	**Audit trail** Notes on each interview were recorded during the interview by the interviewer, and a reflective journal was kept in which the interviewer reflected on how the interviews unfolded and progressed **Reporting of results** Participant demographics were reported. The development of themes was reported in methods, and the quotes to represent each theme were also reported
Dependability	**Audit trail** As mentioned, an audit trail was maintained to document interview progress **Project management** A protocol was developed which detailed the planned methodology and analyses
Transferability	**Sampling** The sampling strategy was purposeful, as participant demographics were monitored during recruitment and oncologists were asked to invite participants with differing characteristics throughout recruitment to try and ensure a diverse range of participants **Reporting of methodology** The COREQ reporting checklist was used to ensure adequate detail concerning the methodology was provided in the manuscript [30]

### Data collection

An interview guide (Supplement A) was developed with input from all investigators and from consumer representatives. Questions were developed to explore individuals’ needs and explore the emotional, mental, spiritual, and social support participants used following their diagnosis. Interviews were conducted by author JN over the phone (*n* = 13), at their home (*n* = 7), or at their treating hospital (*n* = 3). Some participants had their partners present during part of their interviews. The interviewer has experience conducting qualitative interviews with cancer patients and was supervised by two team members with extensive qualitative research experience. The interviewer was a female PhD student who was not involved with participants’ care, and explained the research was part of her PhD.

### Analysis

Interviews were transcribed verbatim and coded in QSR International’s NVivo 12 software. Braun et al.’s [28] methodology for thematic analysis was used to analyse the data. Author JN read and reread the transcripts, coding participants’ experiences using an inductive coding approach. Codes were reviewed within and between transcripts and refined into key themes that described participant experiences. Authors MO and GH reviewed the coding schema and investigator triangulation was used to develop and finalise the coding structure. Discrepancies were resolved via discussion to reach a consensus on the finalisation of codes and themes. Themes were reviewed by all authors. Quotes presented in the results are numbers assigned to each participant’s interview, to deidentify them.

### Rigour

Bradshaw et al.’s [29] measures for establishing credibility, confirmability, dependability, and transferability were adhered to throughout this study (see Table 2). The consolidated criterion for reporting qualitative research checklist (COREQ) informed study reporting [30].

### Ethics

This project received approval from the Sir Charles Gairdner and Osborne Park Health Care Group Human Research Ethics Committee (#RGS3400), St John of God Health Care Human Research Ethics Committee (#1609), and the Curtin University Human Research Ethics Office (#HRE2019-0725). The data that support the findings of this study are available from the corresponding author upon reasonable request.

## Results

Thirty-three eligible participants were identified between February and April 2020, of whom 25 consented. Eight refused participation as they were not interested/did not have time. Two people subsequently withdrew due to a lack of interest in continuing. The remaining 23 participants were interviewed. Demographic information is presented in Table 3.

**Table 3:** Participant characteristics

Participant characteristics (*N* = 23)	*N*	Participant characteristics (*N* = 23)	*N*
Gender		Employment status	
Male	12	Retired	15
Female	11	Unemployed	8
Age (years)		Education status	
Min	22	Primary	1
Max	84	Secondary	18
Mean (SD)	66 (14.7)	Tertiary	4
Diagnosis		Socioeconomic status (SEIFA-IRSD decile)*	
Stage IV lung cancer	6	Min	2
Stage IV pancreatic cancer	2	Max	10
Stage IV CRC cancer	2	Mean (SD)	6 (2.1)
Stage IV cervical cancer	1	Time since diagnosis (months)	
Stage IV glioblastoma	3	Min	2
Metastatic squamous cell carcinoma of the skin	1	Max	86
Stage IV ovarian cancer	2	Mean	24 (25.7)
Stage IV thymic cancer	1	Interview length (minutes)	
Advanced mesothelioma	3	Min	18
Stage IV prostate cancer	2	Max	145
Marital status		Mean (SD)	56.1 (30.2)
Married/de facto	18		
Single/widowed/divorced/separated	5		

^*^Lower Socioeconomic Index for Areas – Index of Relative Social Disadvantage decile indicates greater disadvantage, based on postcode[45]

Participant experiences living with advanced cancer led to the emergence of three key themes: (1) living with a life-limiting diagnosis and uncertainty, (2) living with symptom burden and side effects, and (3) living within the health system, with two subthemes, the patient-clinician relationship, and care coordination.

### Living with a life-limiting diagnosis and uncertainty

All participants were aware they had an incurable cancer diagnosis. They used a variety of methods to cope with their prognosis and the uncertainty; including reframing, milestones, and maintaining autonomy by finding areas of their life they could control. There was a desire for prognostic information from their oncologists, to prepare for the impact of their death on their family. Most participants had been told that they had 3 to 24 months left to live; but a few participants did not know/disclose their prognosis. Not all participants had come to terms with their prognosis.

I’m not happy at all, I got to learn to live with it, but it’s not as easy as people think (P003)

It was particularly hard for participants who had to leave employment, and/or had young adult children.

I actually like working because it took my mind off being sick, I didn’t want to stay at home and be like “poor me, I’ve got cancer” … at least at work I didn’t have to think about it (P010).

Many participants who had been given short prognoses used milestones, such as family events, to reframe the way they viewed the future.

I had set in my mind that I was going to die within six months because they only gave me six months to live … and then one of the nurses said to me: “they tell that to all the stage four cancer patients”. And she said “just fight for as long as you can.” And just by her telling me that, I got it out of my mind and I got a little glimmer of hope that maybe I was going to live past the six months. And when I went past the six months, I started looking forward to the next six months. And I started setting things like my grandchildren’s birthdays….as goals to get to. I’m still doing that now. (P020).

When discussing the nature of their prognosis, some participants used the uncertainty to positively frame their prognosis.

Having a terminal diagnosis, most of the time –no one knows when they’re going to die anyways….you could walk out onto the street and get hit by a car, so most of the time that’s how I think about it. (P015).

A participant who had not received a prognosis wanted to know more from their oncologists, seeking clarity to be able to plan ahead:

I don’t care if he says I’m going to die next week. I’d rather hear that than get some bullshit and then I just drop dead… I’m being a bit sarcastic here, but I want some more clarity and see where I’m going because I’ve got to consider my wife’s situation and … the rest of my life that I’ve got. (P007).

Participants’ experiences coming to terms and living with a terminal prognosis demonstrated that in addition to their personal coping strategies, their family and friends (Table 4) and health professionals have an opportunity to significantly affect their experiences.

**Table 4:** Quotes demonstrating subtheme 3.1.1: living with a life-limiting illness and its impact on others

Description	Representative quotes
Participants had a complex relationship with the support provided by their family and friends. They were aware their terminal diagnosis had a significant impact on their loved ones, but appreciated the support they were able to provide	*I’ve got big family backup … (So if) that (doesn’t) give me reason to get out of bed, nothing does. They’re great, you know. (004)* *The majority of the time, it’s good. Not that its bad, but it’s good that she supports me, and she worries about me, but sometimes you just feel like you’re a burden on everyone and why should she spend her day worried about me, calling over me, getting sick. You know sometimes you feel like a burden on family and friends, It’s nice to have someone who sort of shakes you out of that, I’m sorry for me, and just tries to keep you positive, when you just want to curl up and do nothing, encourage you just to even go out for a drive just to do something, it’s just really, kept me positive, you know when I feel I’ve had enough, just being able to talk to someone who’s just like, yep, yep, look its not fair but let’s just do another round of chemo and see what happens, and do this and do that, so its sort of helped me stay positive and strong. (010)*
Participants greatly appreciated emotional support provided by their friends and family	*It’s just pure support, you know? The other week I was sitting on the lounge, I could hardly move, watching Tarzan. And my mate came over with his wife, and they were sitting in the lounge room there, and we watched Tarzan, the most boring old show, we didn’t even change the channel. And after Tarzan, it was the original Tarzan, so we watched that as well. So it’s just being there for each other, keeping each other company. True blue crap, laughing about the things we missed during Tarzan. No that’s good friendship. That’s support. (014)* *Yeah, he gave up work. Once I got, once I had gone again last year and October, that was where I was having to have, so it was a 21 day cycle, so I’d have to have chemo day 1, chemo again on day 8, and then I’d just have one week off and then back again for it to start again, so that was just – I was really, really sick all the time and he just said “I can’t keep going to work with this” so he stopped working. … And I mean, let’s face it, I don’t know how much time I’ve got left so we’re lucky we can have this together. (021)*

### Living with symptom burden and side effects

A broad range of cancer diagnoses were represented. Symptoms reported included breathlessness, fatigue, diarrhea, vertigo, nausea and vomiting, pain, neuropathy, insomnia, and loss of appetite; and these were attributed to both diagnosis and treatment. Whilst some participants were being seen by community palliative care services, not all had access to these or were unaware of them and their value in managing their symptoms.

Some participants considered the side effects of chemotherapy ‘worth it’, to enjoy quality of life between treatments:

I have to go through it otherwise I don’t give myself a chance at all of beating this or having a delayed end to my life (P007)

One participant commented that seeking help to manage their symptoms following treatment was difficult when experiencing a high symptom burden.

Sometimes when you have this treatment you are not well enough and you can’t make an effort. If somebody rings you, you can say look that’s how I’m feeling, and then the other person can say okay we can do this for you. So you need it coming from outside look we are there if you need us, rather than the patient trying to find who can I ring? Where can I go? That’s hard. (P025).

Some participants were “frightened” by the prospect of engaging with palliative care.

Palliative care was only something I’d ever heard of at the end of people’s lives so that was a frightening few days waiting to go to see the [oncologist and discuss my treatment plan]. (P021).

### Living within the health system

#### The patient-clinician relationship

Participants’ relationships with their clinicians and health professionals were important. One participant acknowledged the power differential created once they received a life-limiting diagnosis.

When this all came about I said that I would throw myself at the feet of the people that know this and deal with this. Give them the best chance to succeed without interfering. (P005).

There was wide variation in participants’ experiences with health professionals. Some participants appreciated a frank and open relationship with their oncologist.

[they’ve] been a good sport, and that’s what I like. It’s up front, it’s honest. Might hurt when you walk away, but that’s how you want it. (P014)

Another participant, whose oncologist referred them to see a psychologist to deal with the anxiety they felt around discussing test results, appreciated that their oncologist was proactive in managing their care and wellbeing.

I didn’t think about [it] at the time, but I realised that [my oncologist] knew that at some stage there wasn’t going to be a good scan and [they] knew I wasn’t going to cope. Bad enough when I’m getting a good scan, what am I going to be like when I get a bad scan?…[They’ve] got great foresight. (P021).

Other participants reported paternalistic attitudes and poor interpersonal communication:

I did not find much joy talking to the doctors. To me, to get better somebody has to show you [they] are caring about you. But I was just another number … if you don’t have any caring attitude from the doctor, you think [they] are not interested in me because [they] think I am going to die in six months’ time. (P025).

Some participants felt excluded from conversations about them and their care, whilst waiting for test results/treatment plans:

When you see them standing ten yards away and they’re talking and they’re looking straight at you, you’re not part of the conversation. Sometimes you’d like to know exactly what they’re talking about. (P032).

These negative or positive experiences were often exacerbated by the coordination of care that participants experienced whilst navigating the health system.

#### Care coordination

Participants who needed to attend multiple hospitals, or multiple departments within a hospital for treatment, indicated they felt that the lack of clear communication between hospitals and health professionals had a negative impact on their care.

I didn’t realise that the hospitals didn’t talk to each other, and as the patient that would be my job … I said to [the urologist], can you ring my oncologist and let them know that I don’t need the tests, and I can start treatment again? And he said “yep”, and I went to the oncologist and I [said] “did they ring you?” And they [said] “no”. And I [thought] here we go again. (P010).

Another participant struggled when their health professionals did not see eye-to-eye about who was responsible for their pain management.

I had to fight to get pain relief. And even then it was between the other GP that I was going to and the urologist, it was like I was the meat between the sandwich. She does it, he does it, she does it… It was an awful position to be in. (P020).

Despite their advanced diagnosis, some participants experienced long delays between getting their diagnosis, being referred and seen by an oncologist. The time from diagnosis to treatment ranged from 2 weeks to 6 months. Issues experienced by participants included needing to see multiple GPs to find someone who could refer them to a clinical trial, or referrals not being followed up.

The surgeon that performed the pleurodesis and told me the diagnosis, [told me to] shake hands and say goodbye. So, that was a very devastating time, it was about 6 months we were in extreme distress … I just couldn’t believe that that was it, so we persisted and asked to be referred to an oncologist. (P033).

Participants whose treatment did not involve multiple hospital departments, or, who had a “*good ally*” in their GP, found navigating the health system easier.

[My GP has] bent over backwards from day one … he seems to have all the right contacts (P007)

Despite seeking care for advanced cancer in a developed country with established care pathways, participant experiences indicate there are substantial gaps in care coordination that need to be addressed.

## Discussion

These findings provide an understanding of participants’ experiences living with advanced cancer whilst engaging with the Australian health system. We found that our key themes of living with advanced cancer and living with symptom burden fit into Arantzamendi et al.’s [31] framework for living well with the awareness of dying. This framework describes living with advanced cancer as an iterative process comprising five phases—struggling, accepting, living with advanced cancer, sharing the illness experience, and reconstructing life—all of which revolve around the central concept of awareness of dying. Our key themes of living with advanced cancer and uncertainty, and living with symptom burden and side effects, reflect these phases and people’s complex and unique experiences. Our participants’ discussion about their symptom burden in the context of this framework illustrates their pervasive awareness of dying and highlights the important role symptom control plays in patient experiences. The role of formal and informal support in participant experiences has been discussed elsewhere [25].

Our results from a heterogenous group of Western Australians living with advanced cancer were similar to those reported by Chambers et al. [22], who examined the experiences of Australian men diagnosed with prostate cancer, with regard to communication and care coordination. These commonalities suggest that the issues patients face navigating the health system may not be diagnosis- or stage-specific. The varied experiences of navigating care indicate an urgent need for additional support and resources at the patient, health professional, and system level.

Participant interactions with their health professionals emphasise the importance of shifting from approaching care using the disease-based biomedical model to patient-centred care. Patient-centred care has been defined as care that is “respectful of and responsive to individual patient preferences, needs, and values and [ensures] that patient values guide all clinical decisions” [32]. It has been recognised that good communication between patients and health professionals is an essential component for eliciting patient values to guide patient-centred care [33]. We found that participants appreciated clear communication with and between their health professionals, and their experiences demonstrate the significant impact patient-clinician communication had on their perceptions of their care.

Patient-clinician relationships in which clinicians proactively referred patients or had time to speak to them about their diagnosis/treatment had a positive impact on participants’ psychosocial wellbeing. Instances in which patients felt no health professionals were taking responsibility for their cancer care or where clinicians did not have time to discuss their diagnosis and treatment regimen were distressing and compounded the negative emotions the patients were already experiencing due to their terminal illness. Poor patient-clinician relationships have been associated with demoralisation in advanced cancer patient populations [34], and greater out-of-pocket expenses [35]. Reasons for a breakdown in communication may include patient-related factors such as health literacy; clinician-related factors such as inter-professional communication issues; and system-related issues, such as lack of time [36]. Patient-reported outcome measures (PROMs) have been recognised as a means to encourage patient-clinician discussion of their symptoms and care, and improve patient-clinician communication [37]. In Australia, The Commission on Safety and Quality in Health Care is currently working to support the implementation and evaluation of PROMs. System-wide implementation of a PROMs’ program within Australia suggests that the program is feasible [38]; however, barriers include the impact on clinical workflow, time for completion inadequate resources, and outcome follow-up [38]. Further research into the implementation and efficacy of PROMs’ programs is necessary to determine if they can bridge gaps in patient-clinician communication.

Participant reports of limited time with clinicians to satisfy their information needs and an inability to access allied health professionals are indicators that staffing and care coordination within the health system need to be assessed. Given that Western Australia has a Cancer Nurse Coordinator Service available to all residents navigating cancer care, ensuring they are adequately resourced is necessary. Previous studies have confirmed the positive impact of nurse-led support programs on cancer care e.g., improved quality of life and reduced hospital readmission rates [39]. Internationally, a systematic review of current literature exploring health professionals’ perceptions of care coordination recommended that health systems needed to (1) develop and provide better educational materials and guidelines for health professionals involved in care coordination, (2) ensure health services have adequate staff, administrative support, technology, and resources to coordinate care, and (3) conduct research to examine current care coordination practices to develop and test tools to help develop shared care plans for patients [40]. Adequate resources for health services are essential for clinicians and staff to be supported to provide well-coordinated care. The recommendations resulting from this study are outlined in Table 5. Further research is necessary to quantify how health services are currently meeting the needs of people with advanced cancer, to ensure existing services are adequately resourced, and to inform future service-level interventions to improve patient care coordination.

**Table 5:** Recommendations to improve patient experiences navigating the health system based on the project’s key findings

Recommendations
The findings from this study highlight several challenges people living with advanced cancer faced whilst receiving treatment. Recommendations and potential solutions to these issues include:
1.Supporting health professionals to improve patient-clinician communication • For example by implementing PROM programs. This would facilitate patients having the opportunity to systematically discuss their symptom burden and any issues they are facing
2.Reviewing the adequacy of staffing for roles that assist in the coordination of cancer care, such as cancer nurse coordinators, patient navigators, and social workers, as all patients are likely to benefit from and deserve access to these professionals for assistance navigating the health system • For example evaluating current cancer navigation programs to determine staff workload and patient needs, in order to determine if additional staff or roles are required to ensure all cancer patients are able to access assistance if they need it.*
3.Further research is necessary to quantify how health services are currently meeting the needs of people with advanced cancer, to ensure existing services are adequately resourced and to inform future service-level interventions to improve patient care coordination • Routine collection of needs data would enable services to monitor and respond to trends in patient needs.* *We recognise there are considerable challenges to implementing such projects; however, these findings support their further development and implementation to improve cancer care experiences of people with advanced cancer

### Limitations

The generalisability of these findings should be considered within the limitations of this study. Whilst data saturation was achieved, recruitment involved clinician referral resulting in recruitment bias. However, due to the wide variation in participant experiences, this bias appears to be minimal. Whilst most participants noted the significant impact of their diagnosis on their carers, the carers were not interviewed as part of this study. Further research examining the experiences of carers and their support needs is necessary. Finally, we note that there was limited discussion of spirituality and its impact on patient experiences. There was only one question in the interview guide asking participants about the role of spiritual support. Given the prominence of spirituality in the experiences of people with advanced cancer in other research, this could be explored further in future research.

## Conclusions

Participant experiences living with advanced cancer are characterised by three key themes: (1) living with a life-limiting diagnosis and uncertainty, (2) living with symptom burden and side effects, and (3) living within the health system. The important role the patient-health professional relationships played in the experiences of people with advanced cancer highlights the value of patient-centred care to support patients with a life-limiting cancer diagnosis. Further attention to the coordination of care for people with advanced cancer is necessary to improve their experiences and the management of their psychosocial burden.

## Supplementary Information

Below is the link to the electronic supplementary material.

Supplementary file1 (PDF 464 kb)

## Data Availability

The data that support the findings of this study are available from the corresponding author upon reasonable request.

## References

[1] Pizzoli SFM et al (2019) From life-threatening to chronic disease: is this the case of cancers? A systematic review. Cogent Psychol 6(1):1577593

[2] White R et al (2021) Treatable but not curable cancer in England: a retrospective cohort study using cancer registry data and linked data sets. BMJ Open 11(1):e040808 33419907 10.1136/bmjopen-2020-040808 PMC7798682

[3] Butow P et al (2015) Clinical pathway for the screening, assessment and management of anxiety and depression in adult cancer patients: Australian guidelines. Psychooncology 24(9):987–1001 26268799 10.1002/pon.3920

[4] Mehnert A et al (2018) One in two cancer patients is significantly distressed: prevalence and indicators of distress. Psychooncology 27(1):75–82 28568377 10.1002/pon.4464

[5] Vodermaier A et al (2011) Disease stage predicts post-diagnosis anxiety and depression only in some types of cancer. Br J Cancer 105(12):1814–1817 22095232 10.1038/bjc.2011.503 PMC3251893

[6] Grassi L, Spiegel D, Riba M (2017) Advancing psychosocial care in cancer patients. F1000Res 6:2083 29259774 10.12688/f1000research.11902.1 PMC5717468

[7] García-Rueda N et al (2016) The experience of living with advanced-stage cancer: a thematic synthesis of the literature. Eur J Cancer Care 25(4):551–569 10.1111/ecc.12523 27297131

[8] Madsen R, Birkelund R, Uhrenfeldt L (2018) Patients experience major changes in life and significant others struggle with caregiving during the course of incurable cancer: a systematic review and meta-synthesis. Eur J Pers Cent Healthc 6(1):88–107

[9] Coyle N (2006) The hard work of living in the face of death. J Pain Symptom Manag 32(3):266–274 10.1016/j.jpainsymman.2006.04.003 16939851

[10] Lin HR (2008) Searching for meaning: narratives and analysis of US-resident Chinese immigrants with metastatic cancer. Cancer Nurs 31(3):250–258 18453882 10.1097/01.NCC.0000305726.72969.07

[11] McCarthy I, Dowling M (2009) Living with a diagnosis of non-small cell lung cancer: patients’ lived experiences. Int J Palliat Nurs 15(12):579–587 20081736 10.12968/ijpn.2009.15.12.45862

[12] Ryan PY (2005) Approaching death: a phenomenologic study of five older adults with advanced cancer. Oncol Nurs Forum 32(6):1101–1108 16270106 10.1188/05.ONF.1101-1108

[13] Sjövall K et al (2011) Experiences of living with advanced colorectal cancer from two perspectives–inside and outside. Eur J Oncol Nurs 15(5):390–397 21163701 10.1016/j.ejon.2010.11.004

[14] Wrubel J et al (2009) End of living: maintaining a lifeworld during terminal illness. Psychol Health 24(10):1229–1243 20204990 10.1080/08870440802320463

[15] Thomas TH et al (2019) Communication differences between oncologists and palliative care clinicians: a qualitative analysis of early, integrated palliative care in patients with advanced cancer. J Palliat Med 22(1):41–49 30359204 10.1089/jpm.2018.0092 PMC6916528

[16] Mosher CE et al (2017) Positive changes among patients with advanced colorectal cancer and their family caregivers: a qualitative analysis. Psychol Health 32(1):94–109 27775432 10.1080/08870446.2016.1247839 PMC5152581

[17] Kitta A et al (2021) The silent transition from curative to palliative treatment: a qualitative study about cancer patients’ perceptions of end-of-life discussions with oncologists. Support Care Cancer 29(5):2405–2413 32918609 10.1007/s00520-020-05750-0 PMC7981304

[18] Fliedner M et al (2019) An early palliative care intervention can be confronting but reassuring: a qualitative study on the experiences of patients with advanced cancer. Palliat Med 33(7):783–792 31068119 10.1177/0269216319847884

[19] Mayland CR et al (2021) A qualitative study exploring patient, family carer and healthcare professionals’ direct experiences and barriers to providing and integrating palliative care for advanced head and neck cancer. J Palliat Care 36(2):121–129 32928058 10.1177/0825859720957817 PMC7961626

[20] Cottingham AH et al (2019) Addressing personal barriers to advance care planning: qualitative investigation of a mindfulness-based intervention for adults with cancer and their family caregivers. Palliat Support Care 17(3):276–285 29880064 10.1017/S1478951518000354

[21] Oosterveld-Vlug M et al (2019) What are essential elements of high-quality palliative care at home? An interview study among patients and relatives faced with advanced cancer. BMC Palliat Care 18(1):1–10 31694715 10.1186/s12904-019-0485-7 PMC6836458

[22] Chambers SK et al (2018) Experiences of Australian men diagnosed with advanced prostate cancer: a qualitative study. BMJ Open 8(2):e019917 29455168 10.1136/bmjopen-2017-019917 PMC5855292

[23] Given, L.M., *The Sage encyclopedia of qualitative research methods*. 2008: Sage Publications.

[24] Van Manen, M (2007) *Phenomenology of practice.* Phenomenology & practice,. **1**(1).

[25] Newton JC et al (2021) The role of psychosocial support in the experiences of people living with advanced cancer: a qualitative exploration of patients’ perspectives. Psychooncology 30(3):287–295 33037707 10.1002/pon.5569

[26] LeBlanc TW, Abernethy AP, Casarett DJ (2015) What is different about patients with hematologic malignancies? A retrospective cohort study of cancer patients referred to a hospice research network. J Pain Symptom Manage 49(3):505–512 25116911 10.1016/j.jpainsymman.2014.07.003

[27] Saunders B et al (2018) Saturation in qualitative research: exploring its conceptualization and operationalization. Qual Quant 52(4):1893–1907 29937585 10.1007/s11135-017-0574-8 PMC5993836

[28] Braun V, Clarke V, Terry G (2014) Thematic analysis. Qual Res Clin Health Psychol 24:95–114

[29] Bradshaw C, Atkinson S, Doody, O (2017) *Employing a qualitative description approach in health care research.* Global qualitative nursing research, **4**. 10.1177/2333393617742282 PMC5703087 29204457

[30] Tong A, Sainsbury P, Craig J (2007) Consolidated criteria for reporting qualitative research (COREQ): a 32-item checklist for interviews and focus groups. Int J Qual Health Care 19(6):349–357 17872937 10.1093/intqhc/mzm042

[31] Arantzamendi M et al (2020) People with advanced cancer: the process of living well with awareness of dying. Qual Health Res 30(8):1143–1155 30539681 10.1177/1049732318816298 PMC7307002

[32] Institute of Medicine (2001) Committee on Quality of Health Care in, A., *Crossing the quality chasm : a new health system for the 21st century / Committee on Quality Health Care in America, Institute of Medicine*, ed. P. Institute of Medicine Committee on Quality of Health Care in America. Content. Washington, D.C.: Washington, D.C. : National Academy Press.

[33] Langberg EM, Dyhr L, Davidsen AS (2019) Development of the concept of patient-centredness–a systematic review. Patient Educ Couns 102(7):1228–1236 30846206 10.1016/j.pec.2019.02.023

[34] Quintero Garzón L et al (2018) Perceived doctor-patient relationship and its association with demoralization in patients with advanced cancer. Psychooncology 27(11):2587–2593 29952046 10.1002/pon.4823

[35] Slavova-Azmanova N et al (2019) How communication between cancer patients and their specialists affect the quality and cost of cancer care. Support Care Cancer 27(12):4575–4585 30927112 10.1007/s00520-019-04761-w

[36] Prouty CD et al (2014) Providers’ perceptions of communication breakdowns in cancer care. J Gen Intern Med 29(8):1122–1130 24599795 10.1007/s11606-014-2769-1 PMC4099451

[37] Yang LY et al (2018) Patient-reported outcome use in oncology: a systematic review of the impact on patient-clinician communication. Support Care Cancer 26(1):41–60 28849277 10.1007/s00520-017-3865-7

[38] Rutherford C et al (2021) Implementing patient-reported outcome measures into clinical practice across NSW: mixed methods evaluation of the first year. Appl Res Qual Life 16(3):1265–1284

[39] Joo JY, Liu MF (2019) Effectiveness of nurse-led case management in cancer care: systematic review. Clin Nurs Res 28(8):968–991 29726271 10.1177/1054773818773285

[40] Hohmann NS et al (2020) Healthcare providers’ perspectives on care coordination for adults with cancer and multiple chronic conditions: a systematic review. J Pharm Health Serv Res 11(2):97–116

[41] Department of Health (2016) W.A., *WA Health Clinical Services Framework 2014–2024*.

[42] Hunter J et al (2019) Coverage of cancer services in Australia and providers’ views on service gaps: findings from a national cross-sectional survey. BMC Cancer 19(1):570 31185937 10.1186/s12885-019-5649-6 PMC6560726

[43] Haynes K et al (2018) Health professionals involved in cancer care coordination: nature of the role and scope of practice. Collegian 25(4):395–400

[44] Monterosso L et al (2016) The Cancer Nurse Coordinator Service in Western Australia: perspectives of specialist cancer nurse coordinators. Aust J Adv Nurs 34:16

[45] Australian Bureau of Statistics. *Table 2 Postal Area (POA) Index of Relative Socio-economic Disadvantage, [2033.0.55.001 Socio-Economic Indexes for Australia (SEIFA), 2016]*. [Internet] 2018 [cited 2020 3 April]; Available from: https://www.abs.gov.au/websitedbs/censushome.nsf/home/seifa2011?opendocument&navpos=260.

