# Supplementary material for: “Who can I ring? Where can I go?” Living with advanced cancer whilst navigating the health system: a qualitative study
Source: Support Care Cancer. 2022 May 10;30(8):6817–26. doi: 10.1007/s00520-022-07107-1 (PMC9213291; doi:10.1007/s00520-022-07107-1)
Supplement: Supplementary file 1 — (PDF 464 kb) [file 520_2022_7107_MOESM1_ESM.pdf]

Supplementary Material

Table S1. Interview Guide used to guide semi-structured interviews.

**Semi-structured interview guide**
General prompts:<br>How do you feel? Could you tell me more about that? How did you manage that? How did that affect you / your family? Can you give me an example of that?<br><br>**1. Please tell me about your experiences following your cancer diagnosis so far**<br>• What treatments have you received?<br>• What impact(s) has it had on you?<br><br>**2. Can you describe the kind of support you have needed in the time since your diagnosis?**<br>***Prompts to be used if dimensions not raised**:*<br>i. How did you cope with the diagnosis?<br>• Can you describe how you have felt, following your diagnosis?<br>• What support did you receive?<br>• Did you need any extra support<br>ii. How do you feel your diagnosis impacted your family and/or friends?<br>• Did they need any kind of extra support?<br>iii. Was there any information you needed following your diagnosis?<br>• What kind of information? When did you need it? Was it readily accessible? What did you find most useful<br>iv. Did your diagnosis and treatment have a financial impact on you and your family?<br>• How did the financial impact affect you?<br>v. Were there any other kinds of support you needed?<br>*Examples if needed: spiritual support, physical support (such as help around the home*).<br><br>**3. Can you describe what kind of support you have used since your diagnosis?**<br>*Prompts to be used depending on responses:*<br>• *If relied on self* – Can you tell me more about how you coped with these needs? How did it affect you?<br>• *Family and friends* – Can you tell me more about how your family and/or friends helped you? What impact did their support have on you?<br>• *Health professionals* – Can you tell me more about how you accessed support from health professionals? Which ones? How did you/ they start the conversation? How did they help you? How did that support affect you?<br>• *Support services* – Can you tell me more about the support services you accessed? How did you find out about them? Were they helpful? How did they help?<br><br>**4. What kind of support(s) helped you the most?**

5. **Did you have any difficulty accessing support(s)?**

6. **If you did need extra support(s), who would you talk to? Where would you go?**
    - Are there any support organisations or services you would access?
    - How did you find out about these services?

7. **Do you feel like you're being supported enough?**

8. **Looking back on your experience so far, do you think that anything(s) could have been done differently?**

End of interview

Thank you for taking time out of your day to participate in this research. That's all the questions I had for you, is there anything else you would like to discuss that hasn't come up today?
